# Maternal and fetal blood lipid concentrations during pregnancy differ by maternal body mass index: findings from the ROLO study

**DOI:** 10.1186/s12884-017-1543-x

**Published:** 2017-10-16

**Authors:** Aisling A. Geraghty, Goiuri Alberdi, Elizabeth J. O’Sullivan, Eileen C. O’Brien, Brenda Crosbie, Patrick J. Twomey, Fionnuala M. McAuliffe

**Affiliations:** 1 UCD Perinatal Research Centre, Obstetrics and Gynaecology, School of Medicine, University College Dublin, National Maternity Hospital, Dublin 2, Ireland; 2 Clinical Chemistry, St. Vincent’s University Hospital, Dublin 4, Ireland; 3 UCD School of Medicine, University College Dublin, Dublin, Ireland

**Keywords:** Pregnancy, Lipids, Cholesterol, References, Cord blood, BMI, Health

## Abstract

**Background:**

Pregnancy is a time of altered metabolic functioning and maternal blood lipid profiles change to accommodate the developing fetus. While these changes are physiologically necessary, blood lipids concentrations have been associated with adverse pregnancy outcomes such as gestational diabetes, pregnancy-induced hypertension and high birth weight. As blood lipids are not routinely measured during pregnancy, there is limited information on what is considered normal during pregnancy and in fetal blood.

**Methods:**

Data from 327 mother-child pairs from the ROLO longitudinal birth cohort study were analysed. Fasting total cholesterol and triglycerides were measured in early and late pregnancy and fetal cord blood. Intervals were calculated using the 2.5th, 50th and 97.5th centile. Data was stratified based on maternal body mass index (BMI) measured during early pregnancy. Differences in blood lipids between BMI categories were explored using ANOVA and infant outcomes of macrosomia and large-for-gestational-age (LGA) were explored using independent student T-tests and binary logistic regression.

**Results:**

All maternal blood lipid concentrations increased significantly from early to late pregnancy. In early pregnancy, women with a BMI < 25 kg/m^2^ had lower concentrations of total cholesterol compared to women with a BMI of 25–29.9 kg/m^2^ (*P* = 0.02). With triglycerides, women in the obese category (BMI > 30 kg/m^2^) had higher concentrations than both women in the normal-weight and overweight category in early and late pregnancy (*P* < 0.001 and *P* = 0.03, respectively). In late pregnancy, triglyceride concentrations remained elevated in women in the obese category compared to women in the normal-weight category (*P* = 0.01). Triglyceride concentrations were also elevated in late pregnancy in mothers that then gave birth to infants with macrosomia and LGA (*P* = 0.01 and *P* = 0.03, respectively).

**Conclusion:**

Blood lipid concentrations increase during pregnancy and differ by maternal BMI. These intervals could help to inform the development of references for blood lipid concentrations during pregnancy.

**Trial registration:**

ROLO Study - ISRCTN54392969. Date of registration: 22/04/2009.

## Background

Pregnancy is a unique physiological state that results in alterations in the mother’s metabolic functioning. These alterations ensure that both the mother and fetus have adequate energy stores throughout pregnancy, and ensure appropriate development of the fetus. As the fetoplacental unit requires glucose, amino acids and lipids throughout pregnancy, the mother’s metabolism must adapt to ensure that this supply is met. These adaptations influence maternal blood lipid concentrations and during pregnancy mothers often enter a physiologically-normal state of hyperlipidemia [1–3]. After delivery, lipid concentrations return to pre-pregnancy concentrations which suggests that this rise in blood lipids could play a role in the physiology of the pregnancy and the development of the fetus [3].

However, raised blood lipid concentrations, above those considered normal during pregnancy, have been associated with negative health outcomes for both the mother and the child. Research suggests that early-pregnancy lipid profiles, particularly elevated total cholesterol and triglyceride concentrations, may be used to identify those at increased risk of developing gestational diabetes [4–6]. Elevated triglyceride concentrations have also been associated with increased risk of pregnancy-induced hypertension and pre-eclampsia in a European cohort [7]. Research has also shown that metabolic changes in pregnancy may be related to the composition of lipids and lipoproteins [8]. This may influence the flux of lipids to the placenta which may, in turn, alter fetal lipid concentrations.

In relation to infant outcomes, maternal triglycerides have been positively associated with fetal size, birth weight and fat mass [9]. Higher total cholesterol and triglyceride concentrations have been associated with higher birth weight [10], increased risk of large-for-gestational age (LGA) [7, 11], and pre-term delivery [7]. However, the point at which maternal blood lipid concentrations become problematic for maternal and infant health outcomes is unclear. One study examined associations of maternal total cholesterol concentrations and fetoplacental endothelial dysfunction and cited above the 75th percentile value (7.5 mmol/L) in their cohort as abnormal, based on observations of endothelial dysfunction above this concentration [12]. There is potential for the use of maternal triglyceride concentrations as predictive factors for LGA births in women with gestational diabetes [13].

Additionally, there is a paucity of data available on fetal blood lipid concentrations. One study identified lipid profiles that differed in small-for-gestational-age infants compared to normal-weight infants, with increased levels of apo C-I–enriched high-density lipoproteins [14], however information is still lacking in this area. Maternal lipid concentrations have been correlated with fetal lipid concentrations at birth among women with diabetes, which highlights the importance of the maternal environment on the fetus during pregnancy [9].

Recommended blood lipid concentrations are available for non-pregnant individuals; below 5 mmol/L for total cholesterol and below 2 mmol/L for triglycerides [15]. However, there are currently no recommended intervals available for maternal blood lipid concentrations during pregnancy or for fetal blood. Thus, the aim of this paper was to address this knowledge-deficit by describing blood lipid concentrations in our cohort of healthy women with singleton pregnancies without gestational diabetes or pre-eclampsia. While these women had all previously given birth to macrosomic infants, the cohort had no complications during pregnancy. As research has shown that blood lipid concentrations differ by maternal BMI [16, 17], intervals were stratified by maternal BMI category.

## Methods

### Study design

A group of 327 mother and child dyads with blood lipid measurements available from during pregnancy from the ROLO (Randomised cOntrol trial of LOw glycaemic index diet) study were included in this analysis. The original study involved 800 secundigravida women, whose first baby had a birth weight above 4 kg, randomised to either a low glycaemic index diet during pregnancy or routine antenatal natal care with no specific dietary advice. This study was carried out in The National Maternity Hospital, Dublin, Ireland, with institutional ethical approval and maternal written consent. Trial registration number for the ROLO study is ISRCTN54392969. Detailed methods and results of the ROLO study have previously been published [18–20]. In brief, the intervention involved one dietary education session (and two follow-up meetings at 28 and 34 weeks’ gestation) with a research dietitian where the women were educated about the glycaemic index and encouraged to follow a low glycaemic index diet. The recommended diet was eucaloric and the women were not advised to reduce their total energy intake. The intervention did not influence birth weight, however, mothers in the intervention group had significantly less gestational weight gain and improved glucose tolerance [21]. The intervention had no impact on maternal or fetal blood lipid profiles so all participants are analysed together in this analysis. The women included all had healthy, full-term singleton pregnancies; there were no adverse maternal outcomes in pregnancy, such as gestational diabetes or pre-eclampsia, and no pre-term births.

### Data collection

Height and weight were measured by trained professionals in early pregnancy (at 13 weeks’ gestation, as calculated based on the participant’s last menstrual period and first trimester ultrasound) in the hospital, and BMI (kg/m^2^) was calculated. Maternal overnight fasting blood samples were taken in early pregnancy and late pregnancy (at 28 weeks’ gestation) and a fetal cord blood serum sample was collected at delivery. Birth weight was recorded at delivery along with incidence of macrosomia (birth weight of or above 4 kg) and LGA, which was classified as birth weight above the 90th centile (taking into account gestational age and sex).

### Laboratory analyses

Serum total cholesterol was measured using Roche cholesterol oxidase method and triglyceride concentrations were measured using the lipase/GPO-PAP (glycerol phosphate oxidase-p-aminophenazone). All measurements were carried out on the cobas c702 module of the Roche Cobas 8000 analyser in accordance with the manufacturer’s instructions (Roche Diagnostics GmbH, Penzburg, Germany). The methods are standardised against the isotope dilution/mass spectrometry (ID/MS) methods.

### Statistical analyses

All variables were evaluated for normal distribution by visual analysis of histograms and non-normal data underwent logarithmic transformation. Antilogarithms were used in the presentation of the results, where required. Intervals were calculated using the 2.5th, 50th and 97.5th centile for the total group and for each BMI category; BMI < 25 kg/m^2^, BMI 25–29.9 kg/m^2^ and BMI ≥ 30 kg/m^2^. Differences between the central tendencies in these categories were examined using one-way ANOVA or Chi-square tests, as appropriate. Correlations between blood lipid concentrations are each time point was examined using Pearson correlation for normally distributed data and Spearman’s correlation for non- normal data. Differences in blood cholesterol and triglycerides based on birth outcomes of macrosomia and LGA were examined using independent sample t-tests, and binary logistic regression was carried out to investigate the association of maternal BMI on this. Statistical analyses were carried out using SPSS (Statistical Package for the Social Sciences) software version 20.0 (IBM, Armonk, NY).

## Results

### Cohort characteristics

Mean BMI at 13 weeks’ gestation was 26.4 kg/m^2^, which is in the overweight category (Table 1). Just over half (54.4%) of the study participants had a BMI ≥ 25 kg/m^2^. Mean infant birth weight was 4.06 kg with 54.7% of infants classified as macrosomic and 29.3% as LGA. Mean birth weight centile was 72.9. There was a significant difference in birth weight with babies born to mothers with a BMI ≥ 25 kg/m^2^ having a higher birth weight than those born to mothers with a BMI < 25 kg/m^2^ (*P* = 0.02).

**Table 1:** Characteristics of mothers and infants from the ROLO Cohort

	Total (*n* = 327)	BMI < 25 (*n* = 149)	BMI 25–29.9 (*n* = 127)	BMI ≥ 30 (*n* = 51)	*P*-value
Maternal Characteristics					
Age at delivery (years)	33.10 (3.90)	33.44 (3.75)	32.80 (4.10)	32.59 (3.91)	0.29
BMI at 13 weeks’ gestation (kg/m^2^)	26.40 (4.60)	22.83 (1.61)	27.11 (1.40)	34.77 (3.65)	–
Achieved 3rd level education (n(%))	174 (58.6)	83 (61.0)	71 (55.0)	18 (40.0)	0.03*
Smoked during pregnancy (n(%))	7 (2.1)	0 (0)	6 (4.7)	1 (2)	0.03*
Ethnicity (Caucasian) (n(%))	323 (98.8)	147 (98.6)	127 (100)	50 (98.0)	0.66
Neonatal/Infant Characteristics					
Male sex (n(%))	157 (47.4)	74 (49.7)	57 (44.2)	25 (49)	0.64
Gestational age at delivery (weeks)	40.4 (1.07)	40.33 (1.07)	40.49 (0.98)	40.40 (1.32)	0.51
Birth weight (kg)	4.07 (0.47)	4.00 (0.47)	4.13 (0.44)	4.10 (0.55)	0.07
Birth weight centile	72.90 (24.9)	74.36 (25.65)	74.27 (22.26)	64.91 (27.91)	0.06

All data expressed as either mean (SD) or n (%) for available data on cohort, *BMI* Body mass index. Statistical comparisons by one-way ANOVA or Chi-square tests. *P < 0.05

The intervals for maternal blood lipids in early and late pregnancy and for fetal cord blood concentrations are described for the total group and each maternal BMI category (Table 2). Both total cholesterol and triglyceride concentrations increased significantly from early pregnancy to late pregnancy (*P* < 0.001). The median concentrations of total cholesterol in early pregnancy, for the total group and each BMI category, are within the recommendations for non-pregnant individuals, below 5 mmol/L, however by late pregnancy 67.7% of the participants exceeded this concentration. The median concentrations for triglycerides in early and late pregnancy are both in-line with the recommendations for a non-pregnant cohort, however, while in early pregnancy 3.9% of the cohort had triglyceride concentrations >2 mmol/L, this increases to 30% in late pregnancy. Early pregnancy cholesterol and triglyceride concentrations were not associated with fetal concentrations, however, in late pregnancy, both total cholesterol and triglyceride concentrations were positively associated with fetal total cholesterol and triglyceride concentrations (*P* = 0.02 and *P* = 0.04, respectively).

**Table 2:** Blood lipid Intervals during early and late pregnancy by maternal BMI category

Early Pregnancy (13 weeks’ gestation)												
	Total Group (*n* = 284)			BMI < 25 (*n* = 136)			BMI 25–29.99 (*n* = 104)			BMI ≥ 30 (*n* = 42)		
Percentile	2.5	50	97.5	2.5	50	97.5	2.5	50	97.5	2.5	50	97.5
Total cholesterol	1.75	4.58	6.73	1.59	4.29	6.69	1.67	4.80	6.92	1.77	4.65	6.31
Triglycerides	0.35	1.03	2.12	0.29	0.94	2.02	0.37	1.07	2.13	0.37	1.32	2.54
Late Pregnancy (28 weeks’ gestation)												
	Total Group (*n* = 293)			BMI < 25 (n = 136)			BMI 25–29.9 (*n* = 114)			BMI ≥ 30 (*n* = 41)		
Percentile	2.5	50	97.5	2.5	50	97.5	2.5	50	97.5	2.5	50	97.5
Total cholesterol	2.1	6.02	8.61	1.83	6.06	8.82	2.41	6.11	8.57	1.97	5.63	8.50
Triglycerides	0.58	1.71	3.24	0.40	1.66	2.91	0.61	1.71	3.33	0.64	1.84	5.12
Fetal Cord Blood (delivery)												
	Total Group (*n* = 227)			BMI < 25 (*n* = 97)			BMI 25–29.9 (*n* = 96)			BMI ≥ 30 (*n* = 33)		
Percentile	2.5	50	97.5	2.5	50	97.5	2.5	50	97.5	2.5	50	97.5
Total cholesterol	1.04	1.70	3.26	1.04	1.73	3.19	1.03	1.68	3.56	0.98	1.63	-^a^
Triglycerides	0.24	0.46	1.45	0.20	0.46	1.38	0.24	0.44	1.51	0.29	0.54	-^a^

All blood lipid concentrations given in mmol/L, *BMI* Body Mass Index (kg/m^2^)

^a^Sample size insufficient to calculate the 97.5th percentile

There were significant differences in blood lipid concentrations between the BMI categories during pregnancy. In early pregnancy, women in the normal-weight category (BMI <25 kg/m^2^) had lower concentrations of total cholesterol compared to women in the overweight category (BMI 25–29.9 kg/m^2^) (*P* = 0.02). Women in the obese category (BMI > 30 kg/m^2^) had higher concentrations of triglycerides in early pregnancy than both women in the normal-weight and overweight categories (*P* < 0.001 and *P* = 0.03, respectively). In late pregnancy, while total cholesterol concentrations didn’t differ significantly between the groups, triglyceride concentrations remained elevated in women in the obese category compared to women in the normal-weight category only (*P* = 0.005). There were no differences in triglyceride concentrations between the overweight and obese categories in late pregnancy. There were also no differences between the cord blood lipid concentrations of infants born to mothers in the normal-weight, overweight or obese categories.

**Fig. 1:**
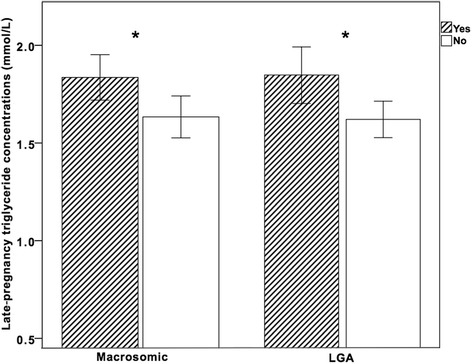
Maternal triglyceride concentrations in late pregnancy in macrosomic neonates (>4 kg weight) or large-for-gestational-age (>90th centile)

Infant size was associated with triglyceride concentrations in late pregnancy in this cohort (see Fig. 1). Triglyceride concentrations were higher in mothers who had a macrosomic baby (1.84 mmol/L vs. 1.63 mmol/L, *P* = 0.01). In cases of LGA infants, triglycerides in late pregnancy were also higher (1.86 mmol/L vs. 1.66 mmol/L, *P* = 0.025). On binary logistic regression, after adjustment for maternal BMI category, triglyceride levels in late pregnancy remained associated with an increased risk of macrosomia (OR: 1.49, CI: 1.05–2.13) and LGA (OR: 1.61, CI: 1.09–2.38). There were no differences in any other blood lipids related to incidence of macrosomia or LGA. Neonatal sex had no association with maternal or fetal lipid concentrations.

## Discussion

Maternal blood cholesterol and triglyceride concentrations increased as pregnancy progressed in this cohort. During pregnancy, maternal blood volume increases by up to 45% (21) and we observed that maternal lipid concentrations increase above this, resulting in a hyperlipidemic state. In addition, maternal weight was associated with blood lipid concentrations as a higher BMI was associated with higher concentrations of total cholesterol in early pregnancy, and triglycerides in early and late pregnancy.

Our results are in accord with previous studies that have shown that blood lipid concentrations increase during pregnancy, with cholesterol concentrations estimated to increase by up to 70% compared to pre-pregnancy concentrations [3, 16, 22–24]. Compared to non-pregnant women, triglyceride concentrations in late pregnancy may be up to 138% higher [3]. While blood lipid concentrations after pregnancy were not available in this cohort, other research has indicated that lipid concentrations return to pre-pregnancy concentrations after delivery, suggesting that the increase in blood lipids could have an important role in the development of the fetus [3, 23, 24]. This is further supported by research highlighting that the increase in blood lipids during pregnancy is not associated with an increased risk of atherosclerosis in women [3, 25]. In fact, raised HDL-C may have a role in protecting the maternal vascular endothelium during pregnancy [26, 27]. It is thought that a failure of the body to increase HDL-C concentrations during pregnancy may be linked with the development of pre-eclampsia [27].

The differences we observed in triglyceride concentrations between the BMI categories could be an indication of altered metabolism or possibly increased levels of insulin resistance in pregnant women with overweight or obesity [28]. Previous research in this cohort reported an association between triglyceride concentrations and birth weight in women in the overweight or obese category only [29]. This highlights the importance of carrying out further research to establish normal lipid concentrations in pregnancy as these concentrations could indicate future risk for the women or offspring. In our cohort there were no differences in fetal lipid concentrations between the BMI groups. Given that maternal concentrations, but not fetal blood lipid concentrations, differed by maternal BMI, this raises an interesting question as to the role of the placenta as a potential mediator. Research has shown that the composition of particular lipoproteins may influence the flux of lipids to the placenta which may play a mechanistic role here [30]. Further research on lipid concentrations in fetal blood may be required to establish normal levels and the physiological implications of abnormal values.

Other studies have reported different blood lipid profiles and metabolic responses in mothers in the normal-weight and overweight or obese categories [17]. One such study found that women in the overweight or obese category had higher baseline cholesterol and triglyceride concentrations compared to women in the normal-weight category; however, in later pregnancy the women in the normal-weight category had higher total cholesterol concentrations as their weekly concentrations increased at a faster rate during pregnancy [16]. This is comparable to our findings where women in the normal-weight and overweight categories had similar total cholesterol concentrations in late pregnancy, despite higher concentrations among overweight/obese categories in early pregnancy. Recently published research has also highlighted how energy metabolism during pregnancy differs between women in overweight and normal-weight BMI categories [30]. During pregnancy women with a BMI > 25 had higher lipid oxidation rates than women with a BMI < 25, particularly at the end of pregnancy [30]. This may also explain why our total cholesterol levels in late pregnancy are more similar between the two groups as the mothers in the overweight category may have had higher lipid oxidation rates at this stage. Published reports describing maternal blood lipids in pregnancy have largely focused on women with gestational diabetes or glucose intolerance [5, 6]. To date, studies relating alterations in blood lipid concentrations during non-diabetic pregnancy have had sample sizes below the 120 recommended by the Clinical and Laboratory Standards Institute [31], or have used values based on just one time point [8, 22, 23].

This analysis had many strengths in that there was a large number of participants in the total study and blood samples were collected in both early and late pregnancy, which can help us understand normal changes over the course of a pregnancy. Blood lipid profiles were also analysed in fetal blood and there is currently a paucity of data in this area. To our knowledge, this is one of the few studies to create lipid reference intervals during pregnancy and to categorise them by BMI, which adds to this body of literature. There were some limitations that must be taken into account. In this cohort, 54.7% of the babies were classified as macrosomic and 29.3% as LGA so this may not be representative of the entire population, however these mothers were healthy with no pregnancy complications. There were also a small number of participants in the obese category (BMI ≥30 kg/m^2^), thus the 97.5th percentile could not be calculated for the fetal cord samples in this group.

With maternal blood lipid profiles in pregnancy being associated with both maternal and child outcomes, it may be advisable to monitor blood lipid concentrations in pregnant women to identify both normal concentrations during pregnancy and the levels at which lipid concentrations become problematic. This could potentially be used to identify concentrations associated with increased risks of adverse outcomes in pregnancy, particularly abnormal fetal growth, and allow for early interventions to prevent negative outcomes for the mother or infant. Given that there was no difference in fetal concentrations between the BMI categories but there was in maternal concentrations, the potential mediating role of the placenta and the influence of blood lipid composition in limiting lipid transfer also remains to be explored.

## Conclusion

Maternal blood lipid concentrations increase over the course of pregnancy and the concentrations at which this can negatively impact both the health of the mother and the child remains to be elucidated. We described intervals in healthy women for total cholesterol and triglyceride concentrations during pregnancy, and also in fetal cord blood. We found that maternal cholesterol and triglyceride concentrations differ by BMI category which may indicate altered metabolic functioning. Blood lipid concentrations are associated with negative outcomes for the mother and infant, thus these intervals can help to inform future references for blood lipid concentrations throughout pregnancy.

## Abbreviations

BMI: Body Mass Index
CI: Confidence Intervals
HDL-C: High-density Lipoproteins
LGA: Large-for-gestational-age
OR: Odds Ratio
